# Not to Be Aligned

**Published:** 1906-07

**Authors:** 


### NOT TO BE ALIGNED.

BECAUSE of the publication of "sixteen pages" of matter from "the other side" and a letter from one of our friends, medically, who did not go into the reorganization of our State Association, there seems to be a slight impression, on both sides, that we are opposed to the organized profession. Had we been able to give the "sixteen pages" more editorial study, it might have been possible to reduce their number. In this matter were references to doctors that were scarcely just, but also claims of unfair treatment by the American Medical Association. All of it made a fine text for the short editorial that preceded it. Further, we must reserve the right to be of, legitimate, aid to those patrons who contribute to the success of this journal.

We published the letter criticizing reorganization because our pages are not sealed to any honest contributor. We are ready to publish letters from either side, to a reasonable amount of space, as well as refutations of any erroneous and injurious charges.

In so far as it is possible, we desired to remain neutral, reserving to ourselves, as owners of the property, only the right to employ the law of self-preservation as it affects our personal and financial interests.

We refuse to allow ourselves to be aligned in this fight, but we hope to be able to see and defend the right as occasion may arise, no matter which side it affects.

There is necessity for reforms, and there is need for conservatism to make the contest for this end successful. Reformers must be fair, those needing reforming must not expect illegitimate help, and neither side must place us in a position that we do not occupy.

H.

---

THE catalogue of the University of Georgia for the year 1906-1907, which is just out, contains an announcement of two courses in forestry. One course will be given in connection with the courses in Political Economy. In this course forestry will be treated from the standpoint of its relation to the welfare of the State and the Nation.

---

BY the use of parallel columns the St. Louis *Medical and Surgical Journal* appears to have proven that the twenty-two cases of death from acetanilid—reported in *Collier's*—were twenty-two deaths from other causes.

---

THE *Journal of the American Medical Association* is quoted as alleging that much of the matter sent out in opposition to its work is false and misleading.

---

THE College of Physicians and Surgeons, San Francisco, expects to have its new building open and ready for work by the beginning of the fall term.

